# Free Access to a Running-Wheel Advances the Phase of Behavioral and Physiological Circadian Rhythms and Peripheral Molecular Clocks in Mice

**DOI:** 10.1371/journal.pone.0116476

**Published:** 2015-01-23

**Authors:** Yuki Yasumoto, Reiko Nakao, Katsutaka Oishi

**Affiliations:** 1 Biological Clock Research Group, Biomedical Research Institute, National Institute of Advanced Industrial Science and Technology (AIST), Tsukuba, Ibaraki, Japan; 2 Department of Applied Biological Science, Graduate School of Science and Technology, Tokyo University of Science, Noda, Chiba, Japan; 3 Department of Medical Genome Sciences, Graduate School of Frontier Sciences, University of Tokyo, Kashiwa, Chiba, Japan; University of Lübeck, GERMANY

## Abstract

Behavioral and physiological circadian rhythms are controlled by endogenous oscillators in animals. Voluntary wheel-running in rodents is thought to be an appropriate model of aerobic exercise in humans. We evaluated the effects of chronic voluntary exercise on the circadian system by analyzing temporal profiles of feeding, core body temperature, plasma hormone concentrations and peripheral expression of clock and clock-controlled genes in mice housed under sedentary (SED) conditions or given free access to a running-wheel (RW) for four weeks. Voluntary wheel-running activity advanced the circadian phases of increases in body temperature, food intake and corticosterone secretion in the mice. The circadian expression of clock and clock-controlled genes was tissue- and gene-specifically affected in the RW mice. The temporal expression of E-box-dependent circadian clock genes such as *Per1, Per2, Nr1d1* and *Dbp* were slightly, but significantly phase-advanced in the liver and white adipose tissue, but not in brown adipose tissue and skeletal muscle. Peak levels of *Per1, Per2* and *Nr1d1* expression were significantly increased in the skeletal muscle of RW mice. The circadian phase and levels of hepatic mRNA expression of the clock-controlled genes that are involved in cholesterol and fatty acid metabolism significantly differed between SED and RW mice. These findings indicated that endogenous clock-governed voluntary wheel-running activity provides feedback to the central circadian clock that systemically governs behavioral and physiological rhythms.

## Introduction

Most mammals exhibit various behavioral and physiological circadian rhythms such as sleep-wake cycles, locomotor activity, feeding behaviour, core body temperature (Tb), blood pressure, immune functions, hormonal secretion and glucose and lipid metabolism that are controlled by endogenous oscillators. The mammalian circadian clock system consists of a master pacemaker in the suprachiasmatic nucleus (SCN) of the anterior hypothalamus and peripheral oscillators in most tissues. Many studies at the molecular level have found that circadian oscillators in both the SCN and peripheral tissues are driven by negative feedback loops comprising the periodic expression of clock genes [1,2]. The environmental light-dark (LD) cycle is the critical time cue for daily resetting of the central clock in the SCN, which entrains the phase of the pacemaker to the environment [1,2]. Peripheral clocks are entrained to the SCN by systemic time cues such as neuronal, humoral and other signals including body temperature [1,2].

A sedentary lifestyle and being overweight due to an imbalance between physical activity and dietary energy intake comprise major public health, clinical and economic issues in current societies [3]. The results of several studies suggest that metabolic conditions affect the circadian systems at the behavioral and molecular levels in mammals [4]. Giving rodents free access to running-wheels that are customarily used to measure circadian activity rhythms alters several aspects of their energy balance including body weight and composition, food intake and energy expenditure, which closely parallels the effects of exercise in humans [5]. Physical exercise is a non-photic time cue of the circadian clock and time-imposed physical exercise entrains behavioral rhythms in rodents [6] and in humans [7]. Bioluminescence studies of tissues from Period2::Luciferase (PER2::LUC) knock-in mice *ex vivo* have shown that scheduled wheel-running affects the phase of PER2::LUC expression in the mouse liver and skeletal muscle [8,9]. On the other hand, free access to a running-wheel as well as metabolic modulation should affect physiological circadian rhythms, although spontaneous movement seems to provide minor feedback to the circadian clock. Free access to a running-wheel shortens the period of the activity rhythm [10] and accelerates re-entrainment to shifted LD cycles [11]. Voluntary wheel-running delays the circadian phase of peripheral PER2 expression in accordance with eating behaviour in PER2::LUC mice [12]. These facts suggest that endogenous rhythmic behavioural activity governed by the circadian system can provide feedback to the master clock in the SCN. The present study assesses the effects of providing mice with free access to a running-wheel on circadian rhythms of feeding, core Tb, plasma hormones such as corticosterone, leptin and ghrelin, in addition to the peripheral expression of clock and clock-controlled genes in mice.

## Materials and Methods

### Animal care and handling

Five-week-old male BALB/c mice (Japan SLC Inc., Hamamatsu, Japan) were habituated with feeding with a normal diet (CE-2; Clea Japan Inc., Tokyo, Japan) *ad libitum* for two weeks under a 12-h light-12-h dark cycle (LD 12:12; lights on at Zeitgeber time (ZT) 0 and lights off at ZT12). A white fluorescent lamp provided 330 lux of light at cage level during the day. The mice were individually housed in cages without running-wheels (SED) to mimic sedentary conditions or with running-wheels (RW), and wheel-running activity was continuously recorded for four weeks using Chronobiology Kit (Stanford Software Systems, Stanford, CA). Locomotor activity was monitored at 5-min intervals and activity data are displayed as actograms, as described previously [13]. Body weight and cumulative food consumption during light and dark periods were assessed weekly. The mice were sacrificed at four-hour intervals to obtain blood and tissue samples. Whole blood was withdrawn under inhalational anesthesia, and the liver, heart, lungs, white (WAT) and brown (BAT) adipose tissue as well as skeletal muscles were dissected, weighed and frozen in liquid nitrogen. All animal experiments were approved by the Animal Care and Use Committees at the National Institute of Advanced Industrial Science and Technology (AIST) (Permission #2013–020) and carried out in accordance with the Guidelines laid down by the NIH in the US regarding the care and use of animals for experimental procedures.

### Monitoring core body temperature

Mice were surgically implanted intra-abdominally with TempDisk TD-LAB data loggers (Labo Support Co. Ltd., Suita, Osaka, Japan) that were programmed to record Tb ± 0.1°C every 10 min. The data obtained from each logger were analyzed using RhManager Ver. 2.09 (KN Laboratories Inc., Ibaraki City, Osaka, Japan).

### Monitoring the timing of food intake

Food intake with or without wheel-running activity was recorded every 10 min using an FDM-300 system and a cFDM-300 system (Melquest, Toyama, Japan), respectively. Data calculated at half-hour intervals were analyzed using Feedam software (Melquest, Toyama City, Japan).

### Measurement of blood hormones

Blood collected in EDTA-coated tubes was immediately separated by centrifugation for 15 min at 5,800 × *g* and then plasma was stored at −80°C. Plasma concentrations of corticosterone, leptin and ghrelin were measured using AssayMax Corticosterone ELISA kits (AssayPro LLC, St. Charles, Missouri, USA), Mouse Leptin ELISA kits (Morinaga Institute of Biological Science Inc., Kanagawa, Japan) and Desacyl-Ghrelin ELISA kits (Mitsubishi Chemical Medience Corporation, Tokyo, Japan), respectively.

### Real-time reverse transcription polymerase chain reaction (RT-PCR)

Total RNA was extracted using RNAiso Plus (Takara Bio Inc., Otsu, Japan). Single-stranded cDNA was synthesized using PrimeScript RT reagent kits with gDNA Eraser (Takara Bio Inc., Otsu, Japan). Real-time RT-PCR proceeded using SYBR Premix Ex Taq II (Takara Bio Inc., Otsu, Japan) and a LightCycler (Roche Diagnostics, Mannheim, Germany) with the primer sequences shown in S1 Table. The amplification conditions were 95°C for 10 s followed by 45 cycles of 95°C for 5 s, 57°C for 10 s and 72°C for 10 s. The amount of target mRNA was normalized relative to that of *Actb*.

### Statistical analysis

All data are expressed as means ± standard error of the mean (SEM). Data from cumulative food consumption were compared between groups using Student’s *t*-test. Other data were statistically evaluated by a two-way analysis of variance (ANOVA) and the Tukey multiple comparison test using Excel-Toukei 2010 software (Social Survey Research Information Co. Ltd., Osaka, Japan). Circadian rhythms were statistically analyzed using the modified cosinor method (nonlinear least-squares (NLLS) Marquardt–Levenberg algorithm) [14]. We defined the function as *f*(*x*) = M + A cos (2π/T(x − ø)) and the variables M (MESOR; mean statistics of rhythm), A (amplitude; half of the total peak-trough variation), T (period) and ø (acrophase) were set as the fit parameters. The circadian period (T) was 24 h under LD 12:12. Acrophase is expressed as elapsed time (h) from 0:00. The significance of circadian rhythmicity was assessed using the zero-amplitude test; *P* < 0.05 was considered evidence of statistically significant rhythmicity for the given period of the cosine curve approximation. Data from cosinor analyses were compared between groups using Welch’s or Student’s *t*-test. *P* < 0.05 indicated a statistically significant difference.

## Results

### Body weight, cumulative food consumption and tissue weight

**Figure 1:**
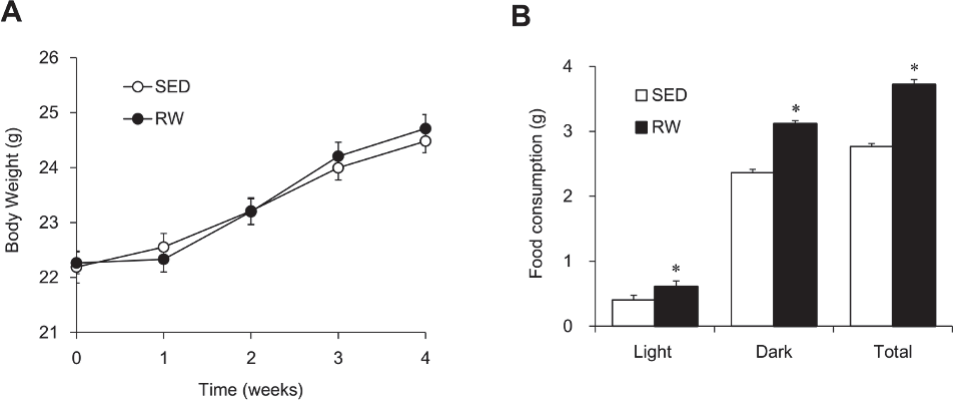
Body weight and cumulative food consumption in sedentary and wheel-running mice. (A) Body weight in mice housed in a sedentary condition (SED; unfilled circles) or given free-access to a running-wheel (RW; filled circles) throughout experimental period. Mice were weighed weekly between ZT2 and ZT3 and data are shown as means ± SEM (*n* = 28). (B) Cumulative food consumption during light (ZT0–12) and dark (ZT12–24) period in SED (unfilled bar) and RW (filled bar) mice. Food consumption by mice was measured daily during the fourth week of access to running-wheels. Data are shown as means ± SEM (*n* = 14). *P < 0.01, significant differences between RW and SED mice. ZT, Zeitgeber time.

We measured body weight gain and cumulative food consumption during light and dark periods, and the tissue weight of SED and RW mice. Free access to a running-wheel did not affect body weight gain during the experimental period (*F*_1, 54_ = 0.13, *P* = 0.714; Fig. 1A). Food consumption was significantly increased in RW mice during both light (ZT0–12) and dark (ZT12–24) periods (light period, *t* = 1.24, *P* < 0.01; dark period, *t* = 0.22, *P* < 0.05; Fig. 1B). The relative food consumption during light period per day was identical between SED and RW mice (14.8 ± 1.9% and 16.7 ± 1.5%, respectively). The relative weights of WAT and BAT to total body weight significantly decreased in RW mice (*t* = 0.03, *P* < 0.01 and *t* = 1.16, *P* < 0.05, respectively), whereas those of the liver, skeletal muscle, heart and lung were similar between SED and RW mice (*t* = 0.85, *P* = 0.579; *t* = 1.03, *P* = 0.959; *t* = 1.39, *P* = 0.114 and *t* = 2.90, *P* = 0.852, respectively; Table 1).

**Table 1:** Relative weight of liver, white and brown adipose tissue, skeletal muscle, heart and lungs of sedentary and wheel-running mice.

Tissue	Group	Relative weight (%BW)
Liver	SED	5.654 ± 0.052
	RW	5.575 ± 0.118
WAT	SED	0.900 ± 0.033
	RW	0.738 ± 0.039**
BAT	SED	0.248 ± 0.008
	RW	0.216 ± 0.010*
Skeletal muscle	SED	0.491 ± 0.005
	RW	0.492 ± 0.014
Heart	SED	0.542 ± 0.030
	RW	0.604 ± 0.022
Lung	SED	0.550 ± 0.008
	RW	0.544 ± 0.026

Values are shown as means ± SEM (n = 6).

*P < 0.05

**P < 0.01, significant differences between SED and RW mice. BW, body weight.

### Circadian rhythms of wheel-running activity, body temperature and food intake

**Figure 2:**
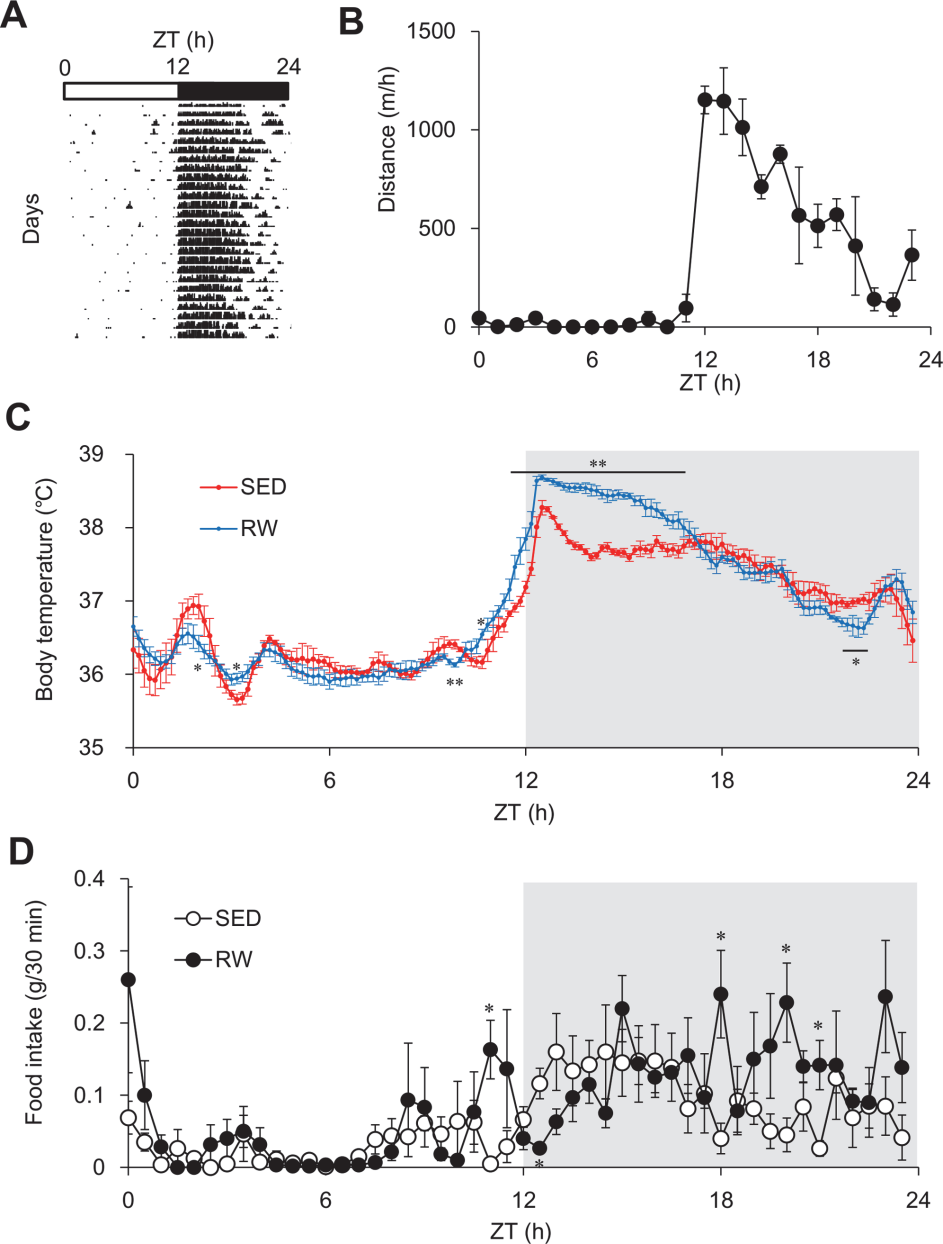
Circadian rhythm of behavioral activity, core body temperature and food intake in sedentary and wheel-running mice. (A) Representative actogram in mice housed with access to running-wheels (RW) for four weeks. Light/dark cycles are shown as white/black bars, respectively. (B) Hourly distance of wheel-running by RW mice over 24 h calculated based on numbers of revolutions/h during fourth week of experiment and running-wheel diameter. Gray shading indicates dark period. Data are shown as means ± SEM (*n* = 3–4). (C) Core body temperature (Tb) rhythm for 24 h under 12 h light-12 h dark cycle. Mice were housed under sedentary (SED; red line) conditions or given free access to running-wheels (RW; blue line) for four weeks and Tb was measured during fourth week. Data are shown as means ± SEM (*n* = 4–5). Gray shading indicates dark period. (D) Circadian rhythm of food intake for 24 h under 12 h light-12 h dark cycle. Food intake was measured every 30 min during fourth week of the experiment in SED (unfilled circles) and RW (filled circles) mice. Data are shown as means ± SEM (*n* = 3–4). Gray shading indicates dark period. *P < 0.05 and **P < 0.01, significant differences between SED and RW mice at corresponding Zeitgeber times (ZT).

Circadian rhythms of behaviour and physiology such as wheel-running activity, feeding and core Tb are regulated by the central pacemaker in the SCN [1]. Wheel-running activity was acutely induced at the light-to-dark transition, peaked during the first half of the dark period, and then gradually decreased to basal levels (Fig. 2A, 2B and S1B Fig.).

Core Tb that acutely increased immediately before the light-to-dark transition fluctuated in a circadian manner in both SED and RW mice (Fig. 2C and S1 Fig.). Significant effects of ZT × running-wheel interaction (*F*_287, 720_ = 4.67, *P* < 0.01) were evident. (Fig. 2C). Core Tb was continuously higher during the first half of the dark period in RW, than in SED mice, which was consistent with the most active period of wheel-running in RW mice (Fig. 2B and 2C). These findings suggest that robust wheel-running activity was involved in the increase in core Tb during this period. Average daily core Tb was identical between SED and RW mice (36.86 ± 0.11°C and 36.98 ± 0.07°C, respectively; *F*_1, 7_ = 3.04, *P* = 0.12). The onset of the increase in Tb was notably about 30 min earlier in RW, compared with SED mice (S1 Fig.).

We evaluated actual food intake by SED and RW mice every 10 min using equipment that rendered real-time bait-box gravimetry possible (FDM-300 and cFDM-300 system; Melquest, Toyama, Japan). Significant effects of ZT × running-wheel interaction (*F*_143, 360_ = 1.89, *P* < 0.01) were evident. (Fig. 2D and S1 Fig.). Food intake significantly increased immediately before the light-to-dark transition and during the latter half of the dark period in RW, compared with SED mice (Fig. 2D).

### Circadian fluctuation of plasma hormone concentrations

**Figure 3:**
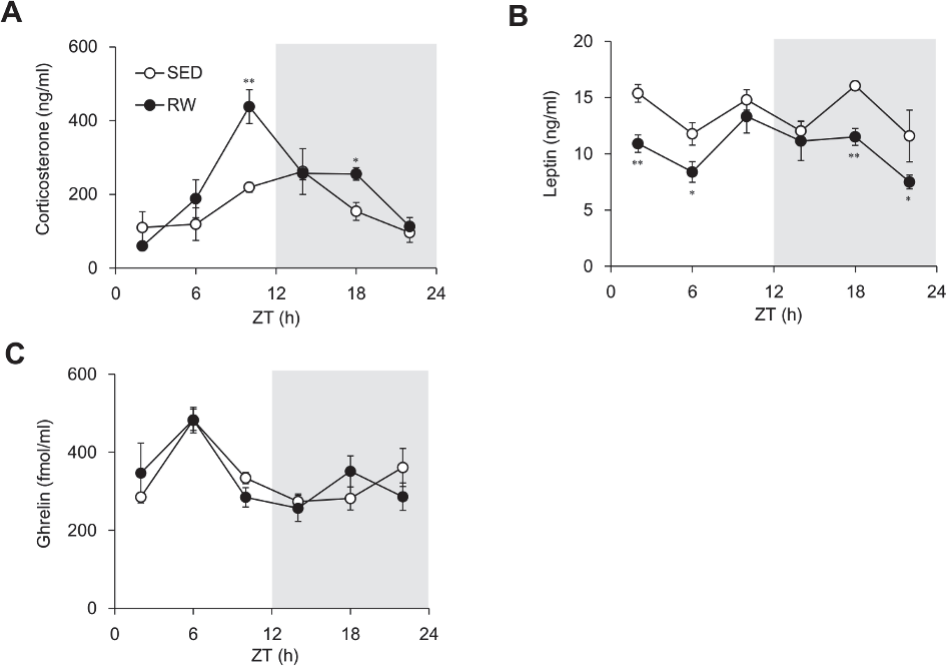
Plasma hormone concentrations in sedentary and wheel-running mice. Plasma corticosterone (A), leptin (B) and ghrelin (C) concentrations at indicated times in mice housed under sedentary (SED; unfilled circles) conditions or given free access to running-wheels (RW; filled circle). Gray shading indicates dark period. Data are shown as means ± SEM (*n* = 4–5). *P < 0.05 and **P < 0.01, significant differences between SED and RW mice at corresponding Zeitgeber times (ZT).

We measured plasma concentrations of corticosterone and the appetite hormones, leptin and ghrelin, every four hours in SED and RW mice. Plasma corticosterone levels peaked at ZT10 in RW mice and were twice as high as those in SED mice, which peaked at ZT14 (*F*_1, 48_ = 8.68, *P* < 0.01; Fig. 3A). This was in accordance with the findings of a previous study showing that plasma corticosterone concentrations at the start of the dark period in mice housed with access to running-wheels for four weeks were twice as high as those in control mice [15]. Plasma leptin levels were significantly down-regulated in RW mice during ZT18 to ZT6 (*F*_1, 48_ = 22.95, *P* < 0.01; Fig. 3B). Wheel-running did not affect the circadian fluctuation of plasma ghrelin concentrations that peaked at the middle of the light period (*F*_1, 48_ = 0.004, *P* = 0.95; Fig. 3C).

### Circadian expression of clock and clock-controlled genes

**Figure 4:**
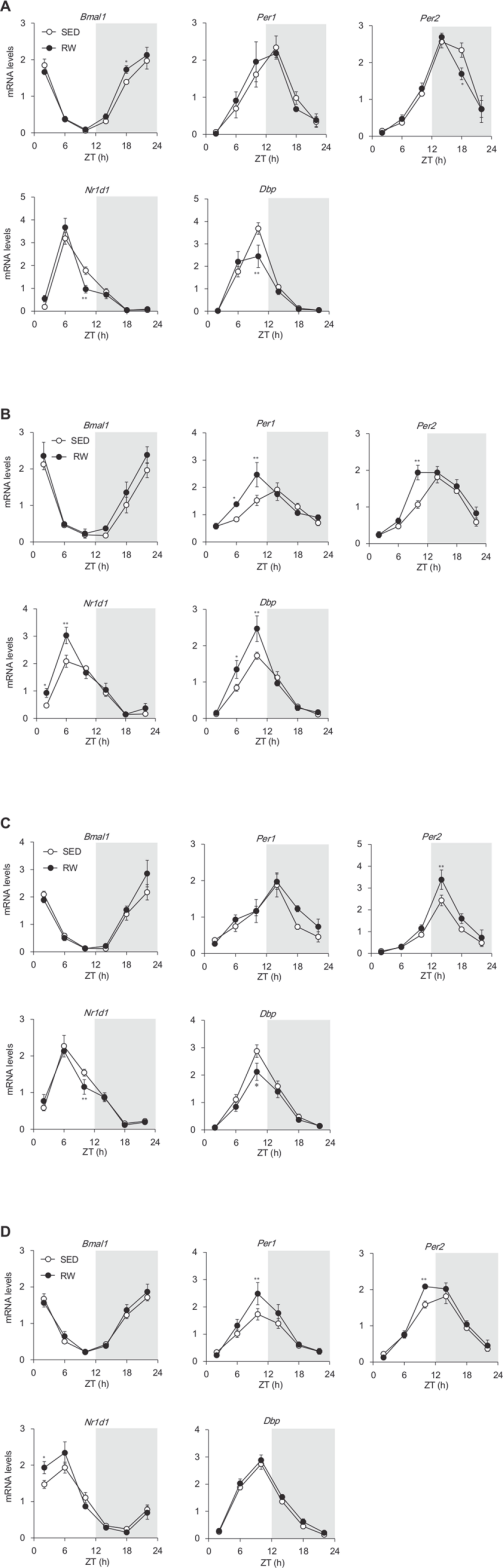
Circadian expression of clock and clock-controlled genes in sedentary and wheel-running mice. Messenger RNA expression of clock and clock-controlled genes in liver (A), white (B) and brown (C) adipose tissue and skeletal muscle (D) from mice housed under sedentary (SED; unfilled circles) conditions or given free-access to running-wheels (RW; filled circles). Gray shading indicates dark period. Data are shown as means ± SEM (*n* = 4–5). *P < 0.05 and **P < 0.01, significant differences between SED and RW mice at corresponding Zeitgeber time (ZT).

We analyzed temporal expression profiles of the circadian clock and of clock-controlled genes in the liver, WAT, BAT and skeletal muscle using a two-way ANOVA and the cosinor method (S2 Table). Chronic wheel-running activity tissue-dependently affected the circadian expression of clock genes (Fig. 4). Cosinor analyses showed that the circadian expression of *Bmal1* was phase-advanced by 0.65 h in the livers of RW mice compared with SED mice (*t* = 2.73, *P* < 0.05; Fig. 4A and Table 2). The mRNA expression of E-box-dependent circadian genes such as *Per2, Nr1d1*, and *Dbp* was also time-dependently affected in the liver of RW mice (*Per2* at ZT18, *F*_1, 48_ = 6.33, *P* < 0.05; *Nr1d1* at ZT10, *F*_1, 48_ = 11.37, *P* < 0.01; *Dbp* at ZT10, *F*_1, 48_ = 16.36, *P* < 0.01; Fig. 4A). The circadian acrophase of *Per1* and *Per2* expression was significantly advanced in WAT by 2.23 and 0.89 h, respectively (*Per1, t* = 4.65, *P* < 0.01; *Per2, t* = 2.88, *P* < 0.05; Fig. 4B and Table 2). Peak levels of *Nr1d1* and *Dbp* mRNA expression were up-regulated, and seemed to be phase-advanced in WAT from RW mice (*Nr1d1* at ZT6, *F*_1, 48_ = 16.01, *P* < 0.01; *Dbp* at ZT10, *F*_1, 48_ = 13.94, *P* < 0.01; Fig. 4B). Cosinor analyses showed that the wheel-running activity did not affect the circadian phase of clock gene expression in BAT, although the levels of mRNA expression were only slightly affected by wheel-running (Fig. 4C and Table 2). The circadian amplitude of *Per1, Per2* and *Nr1d1* expression was significantly increased in skeletal muscle from RW mice (*Per1, t* = 3.85, *P* < 0.01; *Per2, t* = 2.94, *P* < 0.05; *Nr1d1, t* = 3.05, *P* < 0.05; Fig. 4D and Table 2).

**Table 2:** Cosinor analysis of clock gene expression in liver, white and brown adipose tissues and skeletal muscle of sedentary and wheel-running mice.

Tissue	Gene	Group	Mesor	Amplitude	Acrophase (h)
Liver	*Bmal1*	SED	0.99 ± 0.08	1.07 ± 0.11	22.52 ± 0.40
		RW	1.07 ± 0.05	1.11 ± 0.08	21.87 ± 0.26*
	*Per1*	SED	1.00 ± 0.11	1.06 ± 0.16	12.95 ± 0.56
		RW	1.02 ± 0.12	1.08 ± 0.17	12.08 ± 0.62
	*Per2*	SED	1.22 ± 0.11	1.28 ± 0.16	15.37 ± 0.47
		RW	1.16 ± 0.12	1.18 ± 0.17	14.62 ± 0.57
	*Nr1d1*	SED	1.02 ± 0.33	1.41 ± 0.47	7.95 ± 1.27
		RW	1.01 ± 0.45	1.36 ± 0.63	6.85 ± 1.78
	*Dbp*	SED	1.12 ± 0.33	1.67 ± 0.47	9.62 ± 1.07
		RW	0.95 ± 0.21	1.34 ± 0.30	8.96 ± 0.86
WAT	*Bmal1*	SED	0.99 ± 0.13	1.08 ± 0.18	23.45 ± 0.63
		RW	1.20 ± 0.13*	1.24 ± 0.19	23.01 ± 0.58
	*Per1*	SED	1.14 ± 0.04	0.67 ± 0.06	13.41 ± 0.36
		RW	1.36 ± 0.13*	0.81 ± 0.19	11.18 ± 0.89**
	*Per2*	SED	0.94 ± 0.05	0.78 ± 0.07	14.69 ± 0.34
		RW	1.19 ± 0.09**	0.91 ± 0.12	13.80 ± 0.51*
	*Nr1d1*	SED	0.93 ± 0.12	1.05 ± 0.17	7.95 ± 1.27
		RW	1.20 ± 0.23	1.23 ± 0.32	8.39 ± 0.64
	*Dbp*	SED	0.70 ± 0.09	0.81 ± 0.12	10.67 ± 0.58
		RW	0.90 ± 0.19	1.08 ± 0.26	9.75 ± 0.93
BAT	*Bmal1*	SED	1.08 ± 0.08	1.19 ± 0.12	23.11 ± 0.37
		RW	1.18 ± 0.12	1.37 ± 0.16	22.53 ± 0.49
	*Per1*	SED	0.89 ± 0.12	0.65 ± 0.18	12.76 ± 1.03
		RW	1.05 ± 0.10	0.69 ± 0.15	13.79 ± 0.80
	*Per2*	SED	0.88 ± 0.19	0.98 ± 0.27	14.50 ± 1.07
		RW	1.20 ± 0.26	1.42 ± 0.37	14.68 ± 1.00
	*Nr1d1*	SED	0.94 ± 0.16	0.99 ± 0.22	7.86 ± 0.85
		RW	0.87 ± 0.16	0.87 ± 0.22	7.38 ± 0.96
	*Dbp*	SED	1.04 ± 0.20	1.30 ± 0.28	10.75 ± 0.82
		RW	0.83 ± 0.13	0.98 ± 0.19	10.94 ± 0.72
Skeletal muscle	*Bmal1*	SED	0.96 ± 0.06	0.84 ± 0.09	22.69 ± 0.41
		RW	1.01 ± 0.03	0.88 ± 0.04	22.59 ± 0.17
	*Per1*	SED	0.90 ± 0.06	0.73 ± 0.09	10.94 ± 0.46
		RW	1.13 ± 0.12*	1.10 ± 0.17**	10.86 ± 0.60
	*Per2*	SED	0.95 ± 0.05	0.82 ± 0.07	12.59 ± 0.31
		RW	1.08 ± 0.09*	1.03 ± 0.12*	12.51 ± 0.46
	*Nr1d1*	SED	0.98 ± 0.05	0.84 ± 0.07	4.92 ± 0.33
		RW	1.04 ± 0.12	1.12 ± 0.17*	4.50 ± 0.57
	*Dbp*	SED	1.14 ± 0.15	1.29 ± 0.21	9.73 ± 0.63
		RW	1.26 ± 0.15	1.33 ± 0.22	9.86 ± 0.62

Acrophase, delay from 0:00 (lights on); Amplitude, half of total peak-trough variation; MESOR, mean statistics of rhythm. Values are shown as means ± SEM (n = 4–5).

*P < 0.05

**P < 0.01, significant differences between SED and RW mice.

**Figure 5:**
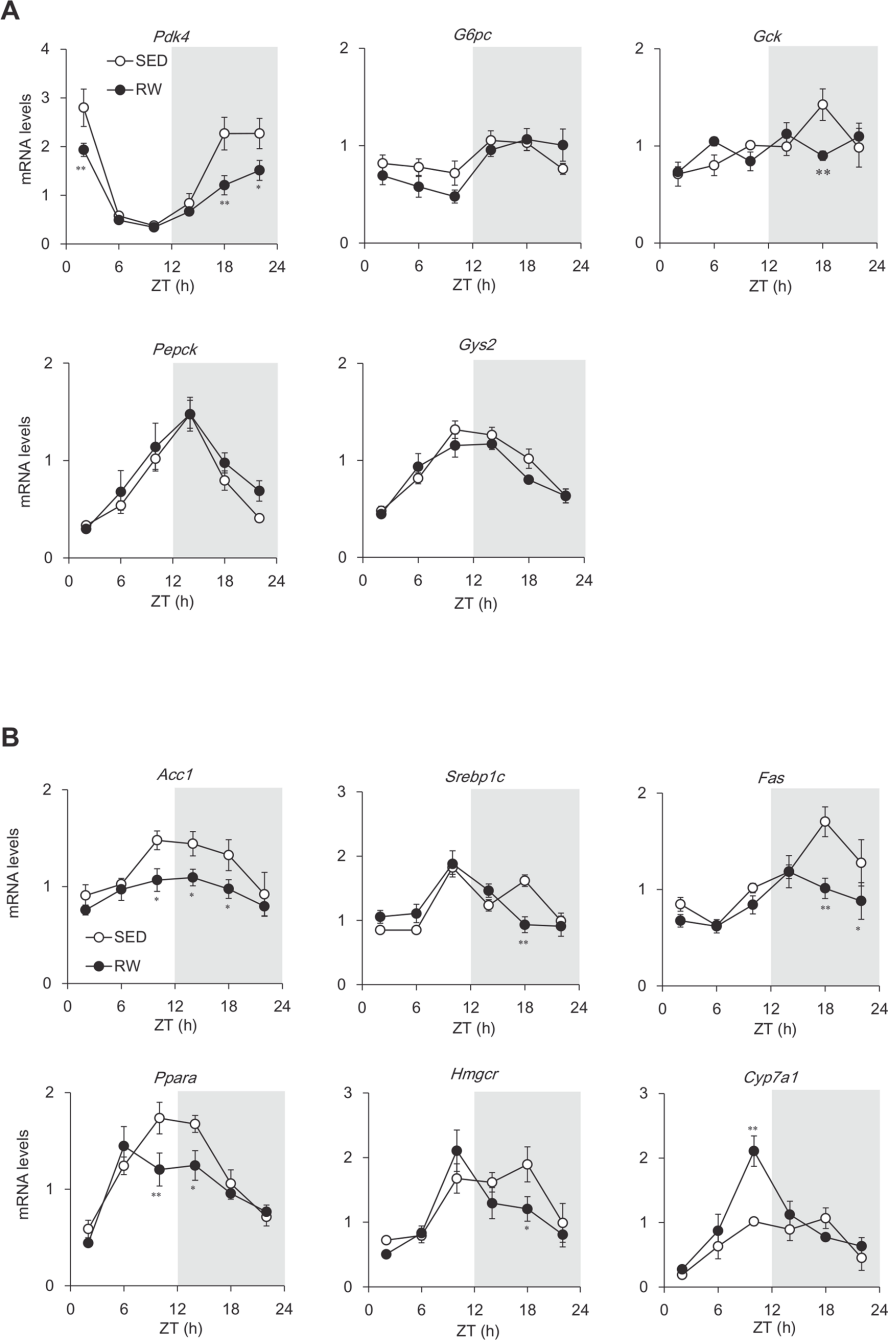
Circadian expression of clock-controlled metabolism-related genes in the liver. Messenger RNA expression of glucose (A) and lipid (B) metabolism-related genes in livers of mice housed under sedentary conditions (SED; unfilled circles) or given free access to running-wheels (RW; filled circles). Gray shading indicates dark period. Data are shown as means ± SEM (*n* = 4–5). *P < 0.05 and **P < 0.01, significant differences between SED and RW mice at corresponding Zeitgeber time (ZT).

We examined the temporal expression profiles of clock-controlled genes involved in glucose and lipid metabolism in the liver (Fig. 5). Chronic wheel-running activity significantly decreased the hepatic mRNA expression of *Pdk4* (that encodes a key enzyme for switching glucose catabolism to fatty acid utilization) at ZT2, 18 and 22. This corresponded to the time of peak expression (ZT2, *F*_1, 48_ = 9.44, *P* < 0.01; ZT18, *F*_1, 48_ = 7.24, *P* < 0.05; ZT22, *F*_1, 48_ = 12.78, *P* < 0.01; Fig. 5A). The mRNA expression of *G6pc, Gck, Pepck* and *Gys2* was essentially identical between SED and RW mice (Fig. 5A). On the other hand, the hepatic mRNA expression of *Acc1, Srebp1c* and *Fas* that encode the key enzymes for fatty acid synthesis, *Ppara* that encodes the key regulator of fatty acid metabolism and *Hmgcr* that encodes the rate-limiting enzyme for cholesterol synthesis, were significantly and time-dependently decreased in RW mice (*Acc1* at ZT10, *F*_1, 48_ = 5.94, *P* < 0.05; ZT14, *F*_1, 48_ = 4.30, *P* < 0.05; ZT18, *F*_1, 48_ = 4.32, *P* < 0.05; *Srebp1c* at ZT18, *F*_1, 48_ = 15.66, *P* < 0.01; *Fas* at ZT18, *F*_1, 48_ = 14.13, *P* < 0.01; ZT22, *F*_1, 48_ = 5.14, *P* < 0.01; *Ppara* at ZT10, *F*_1, 48_ = 9.66, *P* < 0.01; ZT14, *F*_1, 48_ = 6.28, *P* < 0.05; *Hmgcr* at ZT18, *F*_1, 48_ = 5.74, *P* < 0.05; Fig. 5B). Wheel-running strikingly increased peak expression levels of *Cyp7a1* that encodes the rate-limiting enzyme in the synthesis of bile acid from cholesterol (ZT22, *F*_1, 48_ = 22.69, *P* < 0.01; Fig. 5B).

## Discussion

The present study found that chronic free access to a running-wheel affects behavioral and physiological circadian rhythms in addition to the peripheral expression of clock and clock-controlled genes in mice. Temporal profiles of corticosterone secretion, core Tb, food intake and peripheral circadian gene expression were phase-advanced in RW mice, although the effects on gene expression were tissue-dependent. The time at which the increase in core Tb started during the end of light period was significantly phase-advanced in RW, compared with SED mice (Fig. 2C and S1 Fig.). Eating behaviour was significantly evoked at least one hour before the light-to-dark transition in RW mice, although the eating behaviour was gradually evoked after lights-off in SED mice (Fig. 2D and S1 Fig.). These observations suggest that free access to a running-wheel provides feedback to the central clock in the SCN and advances the phase of circadian rhythms in mice. The present observations seem to agree with the findings of previous studies showing that shortening of the circadian period of the sleep-wake cycle [16] and changes in the phase angle [17] are both dependent on wheel-running Another interpretation is that the eating rhythm exerts a phase advancing effect on other rhythms such as peripheral clock gene expression, because food intake is an established and powerful time cue for peripheral clocks and time-imposed daily restrictions of feeding can entrain the peripheral circadian expression of clock genes independently of the master clock in the SCN [18].

The present study found that voluntary wheel-running significantly increased food intake in mice without altering body weight (Fig. 1). The increased appetite in RW mice might result from a continuous decrease in plasma leptin concentrations (Fig. 3C). Chronic exercise reduces plasma leptin concentrations in experimental animals and in humans [19]. As well as suppressing adipose accumulation (Table 1), increased leptin sensitivity might be involved in the reduced concentrations of plasma leptin in RW mice. The temporal expression profiles of hepatic lipid metabolism-related genes, such as *Acc1, Srebp1c, Fas, Ppara, Hmgcr* and *Cyp7a1*, significantly differed between SED and RW mice (Fig. 5B), also suggesting that wheel-running exerts metabolic effects in RW mice.

Many studies have suggested that metabolic conditions affect circadian systems at the behavioral and molecular levels in mammals [20]. High-fat diets attenuate daily feeding rhythms and lengthen the free-running circadian period of locomotor activity [21]. Calorie restriction with ultradian feeding schedules induces phase-advances in wheel-running and body temperature rhythms [22,23]. Bezafibrate-induced activation of PPARα [24,25] and ketogenic diets [26,27] that mimic the metabolic conditions of fasting or caloric restriction without time cues can also advance the phase of circadian behavior, core Tb and hepatic clock gene expression. Various metabolic changes such as those associated with diabetes and obesity tissue-dependently affect peripheral clock gene expression. Both AMPK and the NAD-dependent deacetylase SIRT1 are molecular sensors of the cellular metabolism that controls energy expenditure under caloric restriction and physical exercise [28,29] and they are involved in robust oscillation of the molecular clock in mammals [30]. Lipid metabolism-related gene expression (Fig. 5) and plasma triglyceride and free fatty acid concentrations (S2 Fig.) were significantly affected in RW mice indicating that chronic wheel-running altered energy metabolism. Such changes in cellular metabolic status induced by wheel-running might affect the circadian clocks in this study.

The circadian expression of clock genes was tissue- and gene-specifically affected (Fig. 4 and Table 2). Peak expression levels of E-box-dependent circadian genes such as *Per1, Per2, Nr1d1* and *Dbp* were significantly elevated in a phase-advanced manner in the WAT of RW mice (Fig. 4B). Obesity with adipose accumulation reduces the amplitude of circadian gene expression by decreasing the peak expression of clock genes such as *Per1* and *Per2* in WAT of genetically obese mice [31,32]. Therefore, that adipocyte hypertrophy seems to affect the transcriptional regulation of circadian clock genes as well as that of adipocytokines such as *Lep, Tnf, Ccl2*, and *Adipoq* [33]. A reduction in adipose tissue and/or adipocyte miniaturization induced by chronic wheel-running might be involved in the transcriptional up-regulation of these clock genes.

Peak *Per1, Per2* and *Nr1d1* expression in skeletal muscle was significantly elevated in RW mice. Skeletal muscle adapts to chronic exercise by switching the skeletal muscle fiber type through the expression of contractile protein, myosin heavy chain isoforms, and by an increase in the activity and content of mitochondria, also referred to as oxidative capacity [34]. Chronic exercise-induced activation of PGC1α [35] might be involved in the transcriptional activation of clock genes such as *Bmal1* and *Nr1d1* in skeletal muscle [36]. The increased expression of *Nr1d1* identified in the present study might be an adaptation to chronic wheel-running, because the NR1D1 functions to increase the number of mitochondria in skeletal muscle [37].

Cosinor analyses revealed the phase-advancing effect of wheel-running on the peripheral clock in the liver and WAT but not in BAT and skeletal muscle. The molecular mechanisms regulating peripheral clocks strikingly differ in a tissue-dependent manner [38–40]. Guo et al. [41] reported that tissues differ in terms of their dependence on specific signals that are responsible for enforcing rhythmicity. Parabiotically linking SCN-lesioned and intact mice that shared a circulatory system, resulted in the recovery of rhythmic clock gene expression in the liver and kidney but not in the skeletal muscle, heart and spleen of the lesioned animals [41]. These results suggest that the liver and kidneys rely more heavily on humoral signals than skeletal muscle, heart and spleen to enforce circadian rhythms. Here, voluntary wheel-running affected metabolic status, and the liver and WAT might respond more sensitively to changes in metabolism-related blood-borne signals that can regulate the peripheral clock than BAT and skeletal muscle.

We showed here that voluntary wheel-running for four weeks advanced the circadian phase of peripheral clocks in the liver and WAT in BALB/c mice. However, a recent study of PER2::LUC mice on a C57BL/6J background *ex vivo* found that voluntary wheel-running for two weeks delayed the circadian phase of eating behaviour and hepatic clock-gene expression [12]. These contradictory results of the phase-advancing effect of voluntary wheel-running on peripheral clocks might be a result of differences in experimental periods and/or strains [42]. Furthermore, the *ex vivo* approach to measuring circadian parameters in tissues of PER2::LUC mice has several limitations [43]. The environmental LD cycle can entrain the phase of peripheral clocks independently of the SCN [44]. Therefore, the ability of light to mask peripheral clock gene expression in experiments *ex vivo* should be taken into consideration. Further studies are required to deepen understanding of how voluntary exercise affects the circadian system.

## Supporting Information

S1 Fig Circadian rhythms of food intake, core body temperature and behavioral activity. Circadian rhythms of food intake (blue line), body temperature (red line) and wheel-running (black line) in mice housed under sedentary (SED; A) conditions or given free access to running wheels (RW; B) for four weeks. Results at ZT9–14 show morning nature of RW mice. All parameters were measured every 10 min during week four of a four-week experiment. Gray shading, dark period. Data are shown as means ± SEM (n = 3−4). ZT, Zeitgeber time.
(TIFF)

S2 Fig Plasma metabolic parameters. Plasma tryglyceride (A), free fatty acid (B), total cholesterol (C) and glucose (D) concentrations at indicated times in mice housed under sedentary (SED; unfilled circles) condition or given free-access to running wheels (RW; filled circles). Concentrations were measured using LabAssay Tryglyceride, LabAssay NEFA, LabAssay Cholesterol and LabAssay Glucose kits (Wako Pure Chemical Industries Ltd., Osaka, Japan), respectively. Gray shading indicates dark period. Data are shown as means ± SEM (n = 4–5). *P < 0.05 and **P < 0.01, significant differences between SED and RW mice at corresponding Zeitgeber time (ZT).
(TIFF)

S1 Table Primer sequences for real-time RT-PCR.
(DOCX)

S2 Table Two-way ANOVA of temporal gene expression profiles.
(DOCX)
