# Correction: Free Access to a Running-Wheel Advances the Phase of Behavioral and Physiological Circadian Rhythms and Peripheral Molecular Clocks in Mice

**DOI:** 10.1371/journal.pone.0125646

**Published:** 2015-04-17

**Authors:** Yuki Yasumoto, Reiko Nakao, Katsutaka Oishi

There is an error in the first sentence of the “Monitoring the timing of food intake” subsection of the Materials and Methods. The correct sentence is: Food intake with or without wheel-running activity was recorded every 10 min using a cFDM-300 system and an FDM-300 system (Melquest, Toyama, Japan), respectively.
