# Supplementary figures and images for: Free Access to a Running-Wheel Advances the Phase of Behavioral and Physiological Circadian Rhythms and Peripheral Molecular Clocks in Mice
Source: PLoS One. 2015 Jan 23;10(1):e0116476. doi: 10.1371/journal.pone.0116476 (PMC4304828; doi:10.1371/journal.pone.0116476)

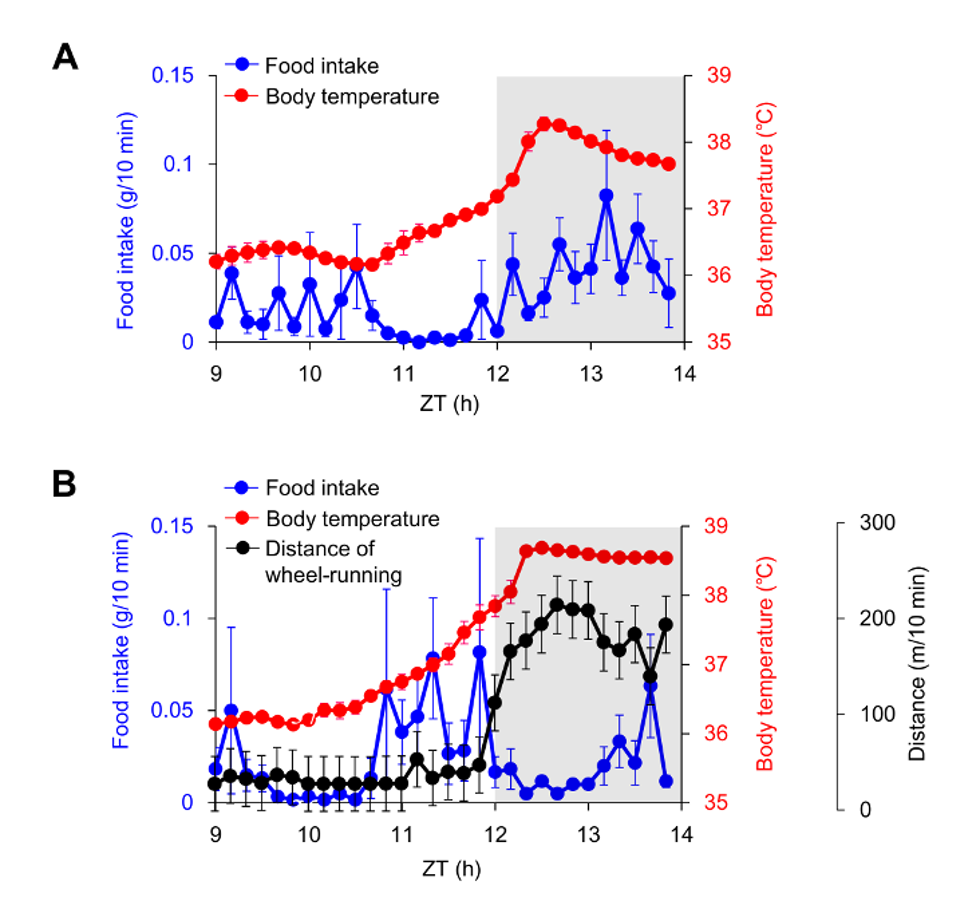

Supplement: S1 Fig — Circadian rhythms of food intake (blue line), body temperature (red line) and wheel-running (black line) in mice housed under sedentary (SED; A) conditions or given free access to running wheels (RW; B) for four weeks. Results at ZT9–14 show morning nature of RW mice. All parameters were measured every 10 min during week four of a four-week experiment. Gray shading, dark period. Data are shown as means ± SEM (n = 3−4). ZT, Zeitgeber time. (TIFF) [file pone.0116476.s001.tiff]

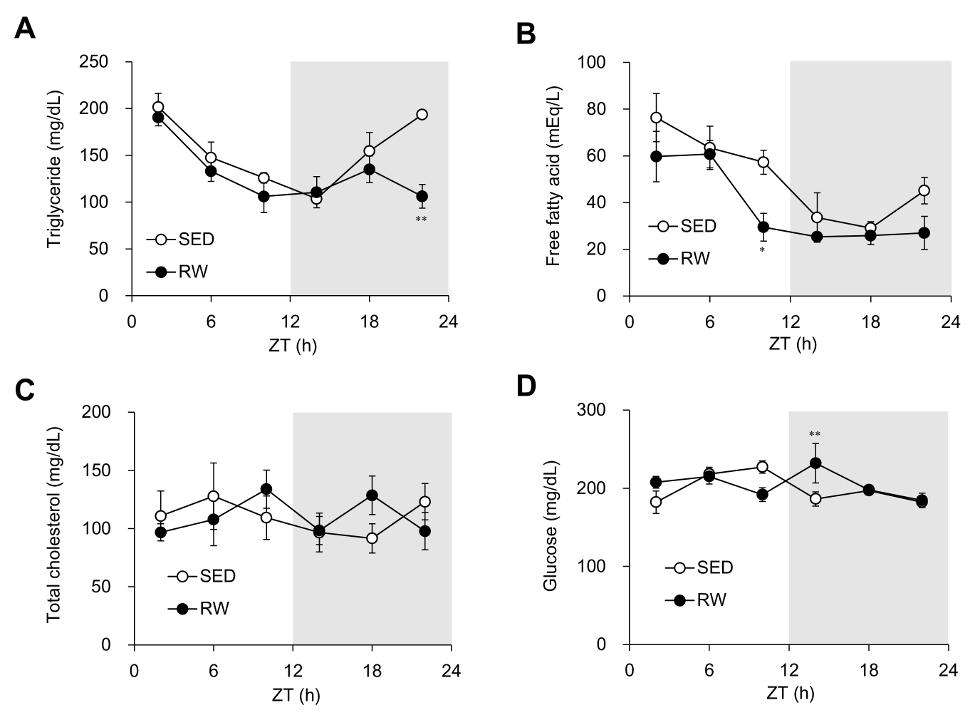

Supplement: S2 Fig — Plasma tryglyceride (A), free fatty acid (B), total cholesterol (C) and glucose (D) concentrations at indicated times in mice housed under sedentary (SED; unfilled circles) condition or given free-access to running wheels (RW; filled circles). Concentrations were measured using LabAssay Tryglyceride, LabAssay NEFA, LabAssay Cholesterol and LabAssay Glucose kits (Wako Pure Chemical Industries Ltd., Osaka, Japan), respectively. Gray shading indicates dark period. Data are shown as means ± SEM (n = 4–5). *P < 0.05 and **P < 0.01, significant differences between SED and RW mice at corresponding Zeitgeber time (ZT). (TIFF) [file pone.0116476.s002.tiff]
