# Supplementary material for: Free Access to a Running-Wheel Advances the Phase of Behavioral and Physiological Circadian Rhythms and Peripheral Molecular Clocks in Mice
Source: PLoS One. 2015 Jan 23;10(1):e0116476. doi: 10.1371/journal.pone.0116476 (PMC4304828; doi:10.1371/journal.pone.0116476)
Supplement: S1 Table — Primer sequences for real-time RT-PCR. (DOCX) [file pone.0116476.s003.docx]

**Table S1. Primer sequences for real-time RT-PCR.**

| Gene | Direction | Primer sequence (5’ to 3’) |
| --- | --- | --- |
| *Bmal1* | Forward | GGCCGAATGATTGCTGAGGAAATCATGG |
|  | Reverse | CTCCGGCTCCAGTACTTCTCA |
| *Per1* | Forward | CCAGATTGGTGGAGGTTACTGAGT |
|  | Reverse | GCGAGAGTCTTCTTGGAGCAGTAG |
| *Per2* | Forward | CACTCAGGAGTGCATGGAGGAGA |
|  | Reverse | CTGCTCTTGCACCTTGACCAGGT |
| *Nr1d1* | Forward | CCCTGGACTCCAATAACAACACA |
|  | Reverse | GCCATTGGAGCTGTCACTGTAG |
| *Dbp* | Forward | GGAACTGAAGCCTCAACCAAT |
|  | Reverse | CTCCGGCTCCAGTACTTCTCA |
| *Pdk4* | Forward | CACATGCTCTTCGAACTCTTCAAG |
|  | Reverse | TGATTGTAAGGTCTTCTTTTCCCAAG |
| *G6pc* | Forward | TGGTAGCCCTGTCTTTCTTTG |
|  | Reverse | TTCCAGCATTCACACTTTCCT |
| *Gck* | Forward | GATCCGGGAAGAGAAGCAAG |
|  | Reverse | GACAGGGATGAGGGACAGAG |
| *Pepck* | Forward | GTGGGCGATGACATTGCC |
|  | Reverse | ACTGAGGTGCCAGGAGCAAC |
| *Gys2* | Forward | CCAGCTTGACAAGTTCGACA |
|  | Reverse | ATCAGGCTTCCTCTTCAGCA |
| *Cyp7a1* | Forward | AGCAACTAAACAACCTGCCAGTACTA |
|  | Reverse | GTCCGGATATTCAAGGATGCA |
| *Ppara* | Forward | tgcaaacttggacttgaacg |
|  | Reverse | aggaggacagcatcgtgaag |
| *Acc1* | Forward | TGCAGGTATCCCCACTCTTC |
|  | Reverse | TTCTGATTCCCTTCCCTCCT |
| *Hmgcr* | Forward | GCAATAGGTCTTGGTGGAGGTGCCAGC |
|  | Reverse | GAAATCATGTTCATCCCCATGGCATC |
| *Srebp1c* | Forward | ATCGGCGCGGAAGCTGTCGGGGTAGCGTC |
|  | Reverse | ACTGTCTTGGTTGTTGATGAGCTGGAGCAT |
| *Fas* | Forward | GGAGGTGGTGATAGCCGGTAT |
|  | Reverse | TGGGTAATCCATAGAGCCCAG |
| *Actb* | Forward | CACACCTTCTACAATGAGCTGC |
|  | Reverse | CATGATCTGGGTCATCTTTTCA |
