# Supplementary material for: Free Access to a Running-Wheel Advances the Phase of Behavioral and Physiological Circadian Rhythms and Peripheral Molecular Clocks in Mice
Source: PLoS One. 2015 Jan 23;10(1):e0116476. doi: 10.1371/journal.pone.0116476 (PMC4304828; doi:10.1371/journal.pone.0116476)
Supplement: S2 Table — Two-way ANOVA of temporal gene expression profiles. (DOCX) [file pone.0116476.s004.docx]

**Table S2. Two-way ANOVA of temporal gene expression profiles.**

| Tissue | Gene | ZT | | Running-wheel | | ZT × Running-wheel | |
| --- | --- | --- | --- | --- | --- | --- | --- |
|  |  | *F*_5, 44_ | *P* | *F*_1, 48_ | *P* | *F*_11, 38_ | *P* |
| Liver | *Bmal1* | 101.80 | < 0.01 | 1.34 | 0.25 | 1.08 | 0.39 |
|  | *Per1* | 25.89 | < 0.01 | 0.02 | 0.90 | 0.49 | 0.78 |
|  | *Per2* | 58.99 | < 0.01 | 0.29 | 0.59 | 1.36 | 0.26 |
|  | *Nr1d1* | 114.89 | < 0.01 | 0.01 | 0.92 | 3.61 | < 0.01 |
|  | *Dbp* | 69.20 | < 0.01 | 1.95 | 0.17 | 3.41 | < 0.05 |
|  | *Pdk4* | 32.92 | < 0.01 | 17.34 | < 0.01 | 2.34 | 0.06 |
|  | *G6pc* | 5.83 | < 0.01 | 1.15 | 0.29 | 1.53 | 0.20 |
|  | *Gck* | 3.79 | < 0.01 | 0.21 | 0.65 | 3.01 | < 0.05 |
|  | *Pepck* | 21.21 | < 0.01 | 2.34 | 0.13 | 0.43 | 0.82 |
|  | *Gys2* | 31.35 | < 0.01 | 2.04 | 0.16 | 1.15 | 0.35 |
|  | *Cyp7a1* | 16.29 | < 0.01 | 7.74 | < 0.01 | 3.96 | < 0.01 |
|  | *Ppara* | 23.28 | < 0.01 | 5.34 | < 0.05 | 2.71 | < 0.05 |
|  | *Acc1* | 5.72 | < 0.01 | 12.58 | < 0.01 | 0.79 | 0.57 |
|  | *Hmgcr* | 12.52 | < 0.01 | 1.85 | 0.18 | 1.68 | 0.16 |
|  | *Srebp1c* | 17.00 | < 0.01 | 0.002 | 0.96 | 4.31 | < 0.01 |
|  | *Fas* | 8.95 | < 0.01 | 10.57 | < 0.01 | 2.08 | 0.09 |
| WAT | *Bmal1* | 45.92 | < 0.01 | 3.31 | 0.08 | 0.32 | 0.90 |
|  | *Per1* | 18.39 | < 0.01 | 4.12 | < 0.05 | 2.85 | < 0.05 |
|  | *Per2* | 48.74 | < 0.01 | 11.10 | < 0.01 | 2.87 | < 0.01 |
|  | *Nr1d1* | 63.22 | < 0.01 | 7.65 | < 0.01 | 2.80 | < 0.05 |
|  | *Dbp* | 61.05 | < 0.01 | 5.59 | < 0.05 | 3.06 | < 0.05 |
| BAT | *Bmal1* | 43.87 | < 0.01 | 0.73 | 0.40 | 1.02 | 0.42 |
|  | *Per1* | 20.45 | < 0.01 | 2.39 | 0.13 | 0.75 | 0.59 |
|  | *Per2* | 46.17 | < 0.01 | 7.01 | < 0.05 | 1.47 | 0.22 |
|  | *Nr1d1* | 69.05 | < 0.01 | 0.78 | 0.38 | 1.06 | 0.40 |
|  | *Dbp* | 73.85 | < 0.01 | 5.96 | < 0.05 | 1.71 | 0.16 |
| Skeletal  muscle | *Bmal1* | 67.97 | < 0.01 | 0.47 | 0.50 | 0.50 | 0.78 |
|  | *Per1* | 31.34 | < 0.01 | 4.64 | < 0.05 | 1.46 | 0.23 |
|  | *Per2* | 90.93 | < 0.01 | 4.68 | < 0.05 | 1.77 | 0.14 |
|  | *Nr1d1* | 56.99 | < 0.01 | 0.65 | 0.43 | 2.11 | 0.09 |
|  | *Dbp* | 202.70 | < 0.01 | 3.96 | 0.05 | 0.15 | 0.98 |
